# Ultra-Nonlinear Subcycle Photoemission of Few-Electron States from Sharp Gold Nanotapers

**DOI:** 10.1021/acs.nanolett.4c03240

**Published:** 2024-08-20

**Authors:** Germann Hergert, Rasmus Lampe, Andreas Wöste, Christoph Lienau

**Affiliations:** † Institut für Physik and Center for Nanoscale Dynamics (CeNaD), Carl von Ossietzky Universität Oldenburg, Carl-von-Ossietzky Str. 9-11, 26129 Oldenburg, Germany

**Keywords:** Ultrafast electron emission, Point-projection electron microscopy, Electron number states, Low energy electrons, Optical near-fields

## Abstract

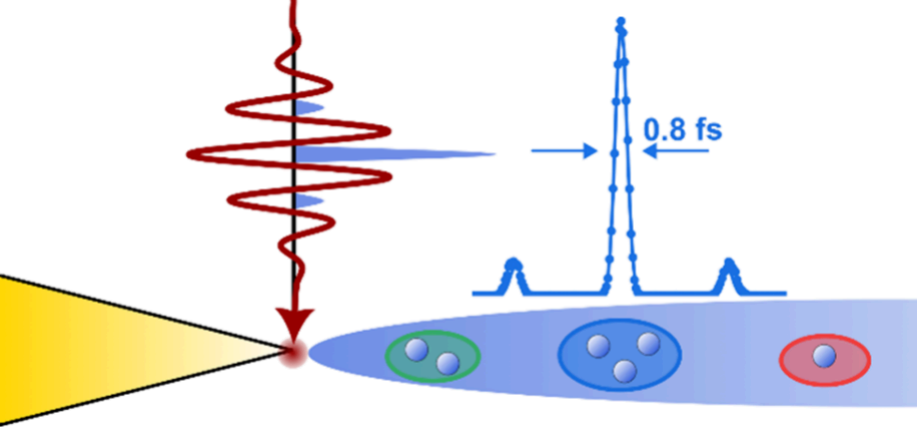

The generation of ultrashort electron wavepackets is crucial for the development of ultrafast electron microscopes. Recent studies on Coulomb-correlated few-electron number states, photoemitted from sharp metallic tapers, have shown emission nonlinearities in the multiphoton photoemission regime which scale with the electron number. Here, we study few-electron photoemission from gold nanotapers triggered by few-cycle near-infrared pulses, demonstrating extreme 20th-order nonlinearities for electron triplets. We report interferometric autocorrelation traces of the electron yield that are quenched to a single emission peak with subfemtosecond duration due to these high nonlinearities. The modulation of the emission yield by the carrier-envelope phase suggests that electron emission predominantly occurs during a single half cycle of the driving laser field. When applying a bias voltage to the tip, recollisions in the electron trajectories are suppressed and coherent subcycle electron beams are generated with promising prospects for ultrafast electron microscopy with subcycle time resolution.

State-of-the-art electron microscopes enable subnanometer spatial resolution and reveal the atomic structure of matter. Advancing such instruments to attosecond temporal resolution may facilitate the direct observation of the motion of charges on their natural scales.^1,2^ This ambitious goal ideally requires the interaction of isolated attosecond electron wavepackets with the sample. Already now, attosecond electron pulse trains^3−5^ have been generated by the quantum-coherent phase modulation of free, swift electrons.^6,7^ Very recently, such pulse trains enabled mapping of subcycle near-field dynamics around metallic nanostructures and inside dielectric resonators.^1,2^

A more direct approach toward attosecond microscopy may rely on the generation of an isolated attosecond beam of low-energy electrons that interacts with a sample in close proximity, e.g., in a point-projection microscope.^8−17^ Nonlinear photoemission from metallic needle sources may in principle generate such pulses and trains of highly coherent femtosecond electron wavepackets for electron microscopy and diffraction have already been demonstrated.^9,18−23^

Emerging studies on few- and many-electron states suggest intriguing new possibilities for further tailoring these wavepackets and their quantum-statistics.^24−27^ Recently, few-electron number states have been generated from nanotapers that exhibit pronounced Coulomb correlations in their kinetic energy spectra.^24,25^ In the multiphoton photoemission (MPP) regime, the nonlinearity of the photoemission yield of higher number states scales directly with the electron number.^24,25^ This can greatly increase the MPP nonlinearity, opening up a window for generating isolated electron pulses with subcycle temporal resolution.

Here, we investigate the generation of few-electron states from a sharp gold taper using few-cycle near-infrared driving pulses. We demonstrate multiphoton photoemission with nonlinearities of up to 20th order for electron triplets. Interferometric autocorrelations of the three-electron yield show single-peak traces with 0.8 fs duration. The sensitivity of the electron yield on the carrier-envelope phase (CEP) of the driving pulses, supported by semiclassical simulations of the emission process, indicates the generation of isolated, subcycle electron pulses in a nondestructive MPP regime. Such ultrafast field-controlled electron pulses may find applications in ultrafast point-projection electron microscopy (UPEM)^8−10,14,16,17,28^ and lightwave electronics.^29−32^

We use passively CEP-stabilized near-infrared pulses with 15 fs full width at half-maximum (fwhm) duration at a wavelength of 2000 nm (∼2.3 cycles) to study the highly nonlinear emission of few-electron wavepackets from a sharp gold nanotaper with ∼25 nm apex radius (Figure 1a). The CEP-stable pulses are generated in a home-built noncollinear optical parametric amplifier with subsequent difference frequency generation operating at a repetition rate of 175 kHz (Supporting Information Section 1). The pulses are focused onto the apex of a gold nanotaper placed inside an ultrahigh vacuum chamber. The pulses are polarized along the tip axis to trigger ultrafast electron emission from the apex. A weak bias voltage of −15 V, corresponding to a static electric field strength of ∼0.1 V/nm, is applied to the tip to accelerate the electrons toward the entrance of a drift tube with an attached time-of-flight delay-line detector (DLD). The DLD provides a full 3D-momentum analysis and, thus, the kinetic energy of each electron (Supporting Information Section 2). The multihit capability of the detector gives this information for multiple electrons in each laser pulse, allowing us to identify the electron number state (here, for *n* = 1–4) of each detection event.^33^

**Figure 1:**
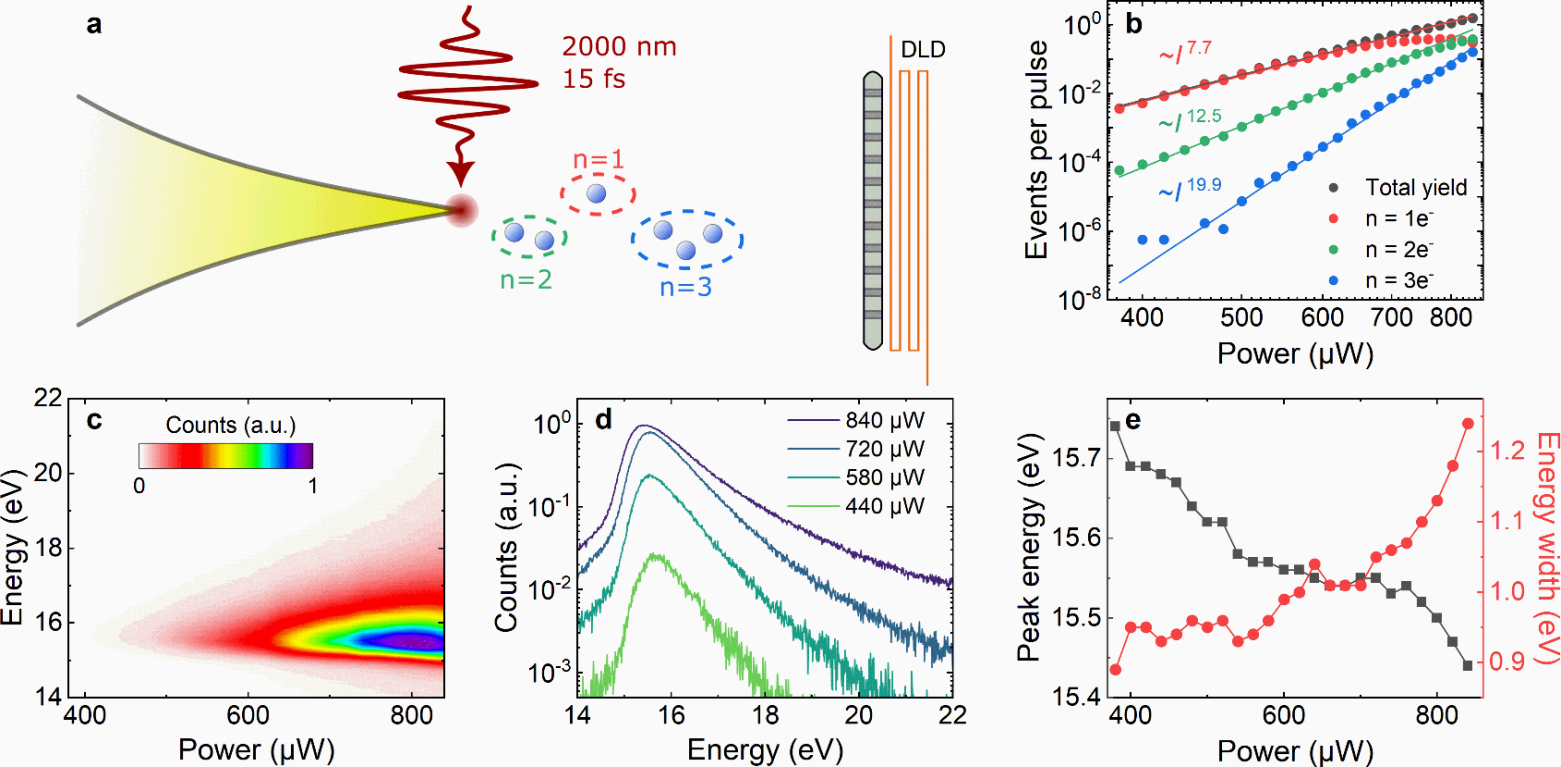
Event-based, highly nonlinear multiphoton photoemission (MPP) from a biased metallic nanotip. (a) Sketch of the experimental setup. Passively CEP-stable, 15 fs near-infrared laser pulses at 2000 nm are focused onto the apex of a gold taper with 25 nm radius. The laser pulses trigger MPP of few-electron wavepackets from the tip apex. The electrons are accelerated by a bias of −15 V toward a time-of-flight delay-line detector (DLD) selecting different electron number states n. (b) Total electron yield as a function of laser power (black dots) on a double-logarithmic scale, revealing a power law of ∼*I*^7.6^. The highest laser power of 840 μW corresponds to a near-field strength of ∼5 V/nm, suggesting MPP with a Keldysh-parameter of γ ≈ 1.6. Power dependencies of the event-selected electrons (circles) show slopes of ∼7.7 (*n* = 1), 12.5 (*n* = 2) and 19.9 (*n* = 3), as indicated by the solid lines. (c) Power-dependent kinetic energy spectra of all detected electrons. (d) Crosscuts through (c) at selected powers. The exponential decays at high energies are characteristic for MPP. (e) Peak energies and widths of the electron spectra, showing a decrease in energy and an increase in width at high powers.

We start by analyzing the total electron yield as a function of the incident laser power (Figure 1b, black dots). A linear fit (Figure 1b, black line) on a double-logarithmic scale reveals a nonlinearity of 7.6 for the electron emission. This value agrees well with the nonlinearity of *s* = (*W* – Δ*W*)/*E*_*P*_ = 7.7, estimated from the work function of bulk gold of *W* = 5.3 eV, the bias-induced lowering of the emission barrier by Δ*W* = 0.38 eV due to the Schottky-effect and average photon energy *E*_*P*_ = 0.64 eV of our pulses. In these experiments, the highest power of 840 μW implies a local field strength of ∼5 V/nm at the tip apex, corresponding to a Keldysh-parameter of γ ≈ 1.6, still within the regime of MPP.^34^ This nonlinearity depends strongly on the number *n* of the electron state, showing the underlying Poissonian statistics as expected for MPP from nanotapers (Figure 1b, colored dots). We observe nonlinearities of 7.7 for *n* = 1, 12.5 for *n* = 2 and even 19.9 for *n* = 3, the highest nonlinearity observed in multiphoton electron emission so far (Figure 1b, colored lines). The order of the nonlinearity approximately scales with electron number as is for an uncorrelated multielectron emission process, governed by Poissonian statistics. A closer inspection of the power-dependent electron yield (see Section 4 of the Supporting Information) reveals large negative values of the Mandel Q parameter^35^ as a signature of sub-Poisson emission statistics of Coulomb-correlated multielectron states.^24,25^

Power-dependent electron spectra are depicted in Figures 1c,d. They show exponential decays toward high energies that are typical for MPP and reflect the kinetic energy distribution of the electron gas inside the tip. The spectra are centered around the applied DC bias voltage of ∼15 eV and their peak position shifts toward smaller energies with increasing laser powers due to ponderomotive interactions with the strong near-infrared field (Figure 1e, black squares).^36^ Additionally, the fwhm of the electron spectra increases from 0.9 to 1.2 eV at average emission rates of 0.01 to 1 electron per laser pulse. Such narrow spectra make our source interesting for electron microscopy.

The observed high nonlinearities make the electron emission sensitive to the temporal shape–and not just the envelope–of the driving electric field, with possible dependence on its carrier-envelope phase. So far, CEP-effects in photoemission have mostly been reported in the strong-field and intermediate regime (γ ≈ 1),^34,37−43^ even though such effects have also been predicted for larger Keldysh parameters.^44^ In these regimes, the CEP allows for controlling the near-field trajectories of the photoemitted electrons and, thus, their kinetic energy spectra.^34,41,45−50^ In the MPP regime, such CEP effects have not yet been observed for metal tips.

In our experiments, the CEP of the driving laser is controlled by inserting a fused silica (FS) wedge pair into its beam path. The measured CEP variation is shown in Figure 2a in the spectrogram (top panel) together with the relative CEP (bottom panel) extracted from a f-2f-interferometer setup.^51,52^ For these experiments, the laser power is set to 680 μW, corresponding, approximately, to a peak near field amplitude of ∼4.5 V/nm and a Keldysh parameter of 1.8. A change of 2π can be observed when inserting ∼66 μm FS, which fits well with the expected thickness of 68 μm required for a 2π shift at 2000 nm. This enables us to controllably vary the CEP and study its influence on the emission yield. The total electron yield (Figure 2b, black dots) shows a clear modulation with the CEP change with an amplitude of ∼6% and a periodicity of about 2π. For the three-electron events (Figure 2b, blue dots), we observe a more pronounced modulation of ∼18%, again with 2π-periodicity. We see that event rate and CEP modulation decrease with increasing FS insertion. This can be explained by a finite temporal stretching of the pulse to up to ∼20 fs with increasing FS thickness. As guides to the eye, the dashed black and blue lines in Figure 2b show a CEP modulation with exactly 2π period, following the descending trend of the measured data.

**Figure 2:**
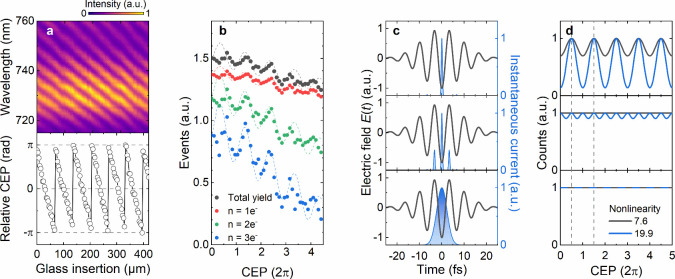
Dependence of ultrafast few-electron emission on the carrier envelope phase (CEP). (a) Top panel: Interferogram obtained by a controlled CEP variation via insertion of a fused silica wedge pair in a f-2f-interferometer. Bottom panel: Extracted relative CEP change from the interferogram. (b) Measured CEP-dependence with ∼2π period of the total electron yield (black circles) and the yield of the *n* = 1, 2, 3 events (red, green and blue circles, vertically shifted for clarity). A strong modulation of up to 18% (*n* = 3) can be seen, indicating subcycle electron emission. The experiments have been performed at a laser power of 680 μW, corresponding to a peak near field amplitude of ∼4.5 V/nm and a Keldysh parameter of γ = 1.8. (c) Simulated instantaneous emission current (blue) during a short pulse (black) for three different analytical models for unidirectional (top), bidirectional (middle) and envelope-driven (bottom) emission. (d) Calculated CEP-dependencies for the different emission models in (c) with nonlinear order of 7.6 (black) and 19.9 (blue), using a 15 fs pulse at 2000 nm. Only the first, unidirectional model, assuming emission during negative half-cycles, shows a pronounced CEP-dependence with a period of 2π (dashed lines).

Such a CEP-dependence suggests an instantaneous nonlinear electron emission process that follows the electric field of the laser pulses, rather than the intensity envelope. To rationalize these findings, we compare three different analytical emission models in Figure 2c (blue areas). In all cases, we assume bandwidth-limited few-cycle near-infrared pulses at 2000 nm with variable CEP (black line). The fwhm of their intensity profile is 15 fs. The first model (Figure 2c, top panel) assumes an emission current *C*(*t*) that follows the electric field *E*(*t*) of the laser only during negative half-cycles, with *C*(*t*) ∼ *E*(*t*)^2*s*^ θ[-*E*(*t*)]. Here θ denotes the Heaviside-function and *s* = 7.6 is the nonlinearity of the photoemission. This model describes a unidirectional emission in a field-driven picture. Emission from the tip occurs only during those half-cycles in which the local near field pushes the electrons toward the vacuum. Alternatively, we consider a bidirectional model. Here, the emission also follows the laser field but is now allowed during both half-cycles (Figure 2c, middle panel), *C*(*t*) ∼ *E*(*t*)^2*s*^. Finally, an envelope-driven emission model (Figure 2c, bottom panel) assumes an electron yield that follows the field envelope, *C*(*t*) ∼ ⟨*E*(*t*)^2^⟩^*s*^,and not the instantaneous field. Here, ⟨···⟩ denotes a cycle average.

These three models result in substantially different CEP-dependencies of the total emission yield ∫_–∞_^∞^*C*(*t*)d*t*, which is the quantity that is measured in our experiments. In Figure 2d, we display the CEP-dependencies for each of the three models. The simulation parameters are taken from Figure 2c and only the CEP is varied. In the simulations, we use emission nonlinearities of *s* = 7.6 (black) and *s* = 19.9 (blue), corresponding to the measured nonlinearities of the total electron yield and the three-electron number state in Figure 1b. The first, unidirectional emission model (Figure 2d, top panel) shows a strong CEP-modulation of 18% and 76% respectively and a CEP-periodicity of 2π. In the bidirectional model, a weak CEP-dependence remains, but now with a periodicity of π (Figure 2d, middle panel). The CEP-dependence vanishes in the envelope-driven model (Figure 2d, bottom panel). Only the unidirectional emission is in qualitative agreement with our experiments since it predicts a finite CEP effect with a periodicity of 2π. The two other models cannot account for our observations. We presume that this reduction in contrast results from finite long-term CEP fluctuations of our experimental setup. The simulations for the unidirectional emission (Figure 2c, top panel) suggest that most of the electrons that are photoemitted from the tip are born in a small fraction of the central cycle of the driving pulse.

To analyze these emission dynamics experimentally, we use interferometric autocorrelation measurements. For this, a Michelson interferometer is employed that splits the laser into a phase-locked pair of two identical pulses with variable time delay τ. The power of the pulse pair at τ = 0 fs is set to 620 μW, such that the *n* = 1 events show a nonlinearity of ∼7.7, independent of τ. By varying τ, we record energy-resolved electron autocorrelations (EAC) for electron number states between *n* = 1 and *n* = 3. We measure the kinetic energy of each detected electron and record the electron number state *n* for each laser pulse. The resulting energy-resolved EAC for the total electron yield is depicted in Figure 3a while the energy-resolved EACs are displayed, for different *n*, in Figure 3b-d. The corresponding energy-integrated EACs are depicted in Figure 3e-h (black and colored dots). A zoom into the central peaks is shown in the insets. Due to the low event rate of ∼10^–4^ per pulse for the electron triplets, the EACs are averaged over multiple EAC traces to reach a good signal-to-noise ratio. The reported data are composed of ten separate measurements performed within 5 h. Since our laser shows CEP drifts on a time scale of tens of minutes (Supporting Information Section 1), the EACs average over many CEP phase angles.

**Figure 3:**
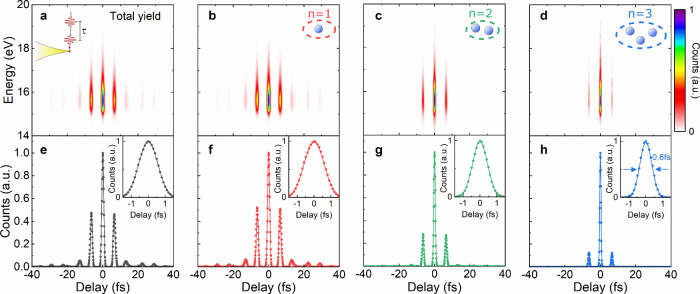
Ultrafast energy-resolved and event-selected electron autocorrelation (EAC) traces. The 15 fs pulses at 2000 nm are split into identical pairs with variable delay τ and are focused onto the tip. The peak power is set to ∼620 μW at τ = 0 fs, giving MPP of *n* = 1 electrons with a slope of 7.7. (a) EAC trace of the total electron yield (b–d) Event-selected electron spectra as a function of electron number *n* and pulse delay. For higher number states, most of the electron emission is observed during the central peak around τ = 0 fs. The kinetic energy distribution shows no significant change with *n*. (e–h) Event-selected EACs, integrated over the kinetic electron energy (filled circles). The EACs show strong electron emission predominantly for delays around τ = 0 fs. For the three-electron events, the width of the central peak is only 0.8 fs. The solid lines through the data show simulated EACs, assuming an instantaneous, unidirectional emission model and using the measured electric field pulse profile. The nonlinearities in the emission model are set to 6.5, 6, 11 and 17.

The EAC recorded for the total electron yield shows significant electron emission for delays of up to τ = ± 10 fs. Due to the high photoemission nonlinearity, a large yield requires constructive interference between the two pulses and thus occurs mainly for delays of integer multiples of the optical period. Almost the entire emission is confined to the central peak and its first sidepeaks, while higher sidepeaks are largely suppressed. The energy spectra in Figure 3a are slightly broader for the central peak than for the sidepeaks. Their fwhm decreases from 1.2 eV for the central peak to ∼0.9 eV for the sidepeaks. For *n* = 1–3 the spectra (see Section 3 of the Supporting Information) show a monoexponential decay at large energies, pointing to photoemission from a heated electron gas^53^ while clear signatures of ponderomotive broadenings are absent. This points to an enhanced heating of the electron gas with increasing pulse energy. Consistent with the results in Figure 1, the spectral width decreases from 1.2 eV for the central peak to ∼0.9 eV for the sidepeaks. The energy-integrated EAC in Figure 3e emphasizes the pronounced temporal confinement of the total yield to delays of constructive interference. For the central peak, the width of the autocorrelation is less than 1.4 fs (see inset). This autocorrelation is quantitatively reproduced by taking the independently measured electric field profile *E*(*t*) of the laser (Supporting Information, Section 1) and a nonlinear order of *s* = 6.5. In the simulation, shown as a solid line in Figure 3e, unidirectional emission is assumed, *C*(*t*) ∼ *E*(*t*)^2*s*^ θ[-*E*(*t*)]. Since the CEP of the laser was not kept constant during the measurements, we include phase-cycling into the analytical model, averaging the EACs over four different CEPs (0,π/2,π,3π/2) of the total laser waveform.

Energy-resolved EACs for the three investigated number states are deduced by sorting each detection event (Figure 3b-d). The electron spectra for *n* = 1–3 have a similar energy distribution. The most pronounced difference between these EACs is the strong suppression of side peaks along the delay axis. While eight side peaks are seen for *n* = 1, the emission is mostly confined to the central peak for *n* = 3. The energy-integrated EACs in Figure 3f-h emphasize not only the side peak suppression, but importantly also a strong reduction in the temporal width of the central peak with increasing *n*. The fwhm of this central peak decreases from 1.4 fs for *n* = 1 to 0.8 fs for *n* = 3 (see insets in Figure 3f-h). Thus, the EAC for the three-electron number state mainly shows a single emission peak of sub-fs width. The observation of such a narrow peak alone is not sufficient to draw conclusions about the pulse duration of the photoemitted electrons since EACs do not give direct insight into the electron emission dynamics. Together with the information about the unidirectional emission obtained from the CEP studies, this gives evidence for the emission of an, essentially, isolated burst of subcycle electrons from our tips. To support this conclusion, we first show that the energy-integrated EACs for different *n* are well reproduced by the unidirectional emission model if phase cycling and the high nonlinearity of the instantaneous electron current are included. The nonlinearities of the emission model are set to *s* = 6.5 (total yield), 6 (*n* = 1), 11 (*n* = 2) and 17 (*n* = 3) to reach an optimum match with experiment. The simulation results are shown as solid lines in Figure 3f-h and in the corresponding insets.

To rationalize the experimental observations, we analyze MPP from a biased metal surface by numerically solving a one-dimensional time-dependent Schrödinger equation (TDSE), *iℏ∂*_*t*_ ψ(*x*, *t*) = *H*(*x*, *t*)ψ(*x*, *t*), for a single electron with wave function ψ(*x*, *t*).^47^ The electron is initially bound inside the metal, modeled by a half-infinite quantum well *V*_tip_ (*x*), with a depth of 10.8 eV and a width of 80 nm. The potential contains 429 bound states with a largest energy spacing of 50 meV. This forms a quasi-continuum of states. Initially, the electron is set in a single eigenstate with an energy of *W* = −5.3 eV, close to the work function of gold.

To mimic the experiment, the electron interacts with a near field *E*_NF_ (*x*, *t*) = *F*_0_ α(*x*) *E*_*t*_ (*t*), induced by a laser with field strength *F*_0_ = 0.25 V/nm, center wavelength 2000 nm and a Gaussian profile *E*_*t*_ (*t*) with 15 fs fwhm and variable CEP. The spatial near-field profile
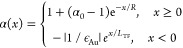
is confined to a decay length of R = 25 nm outside the metal and has a field enhancement of α_0_ = 8,^42^ giving a maximum field amplitude of 2 V/nm. This corresponds to γ = 3.7, well within the MPP regime. The field component inside the tip is strongly reduced by the dielectric constant of gold^54^ at 2000 nm, ϵ_Au_ = −189 + *i*25, and screened^55^ within *L*_TF_ = 0.5 Å. Spatial integration of the field yields the interaction potential *V*_NF_ (*x*, *t*) = *e*∫_*-∞*_^*x*^*E*_NF_ (*x*′, *t*)d*x*′ (*e*: elementary charge). An additional bias potential *V*_DC_ (*x*, *t*) results in a static field outside the tip that pushes the electrons away from the surface. The spatial variation of *V*_DC_ (*x*, *t*) is obtained from the analytical solution of the Poisson equation for a hyperbolic tip shape in front of a 1 cm distant counter electrode.^56^ This results in a total Hamiltonian of *H*(*x*, *t*) = −*ℏ*^2^∂_*x*_^2^/(2*m*) + *V*_tip_(*x*) + *V*_NF_(*x*, *t*) + *V*_DC_(*x*, *t*).

We start by simulating the electron density ρ(*x*, *t*) = |ψ(*x*, *t*)|^2^ that is photoemitted from the tip in the absence of a bias voltage (Figure 4a). The driving laser field is depicted by the black line. The density profile ρ(*x*, *t*) in the vicinity of the tip shows that electron emission is confined to the field maxima of each negative field cycle, generating a force that pushes the photoemitted electrons away from the metal. In the absence of a bias, the quiver motion and recollisions of the photoemitted electrons with the metal surface play a profound role for the emission dynamics. This is clearly seen when calculating the instantaneous emission current *C*_inst_ (*t*) = ∂_*t*_ ∫_*x*_0__^∞^ρ(*x*, *t*)d*x* (black line in Figure 4b). The current is evaluated for distances exceeding *x*_0_ = 1.7 nm. Negative values of *C*_inst_ (*t*) imply that electrons are pushed back toward the metal during positive half cycles. Importantly, the recolliding electrons that are generated in one cycle can interfere with the newly born electrons in the subsequent cycle. The resulting interferences are seen in the spatiotemporal emission pattern ρ(*x*, *t*) and as marked subcycle structures in *C*_inst_ (*t*). These interpulse interferences have a pronounced effect on the temporal dynamics of the electron beam that is generated by MPP.^57,58^ Already after a short propagation of *x*_*d*_ = 20 nm, the temporal profile of the free electron beam ρ(*x*_*d*_,*t*) (red line in Figure 4b) has increased to more than 50 fs. Recollisions, and the resulting intercycle interferences, result in a strong spectral broadening of the generated free electron pulse and–thus–pronounced dispersion upon propagation.

**Figure 4:**
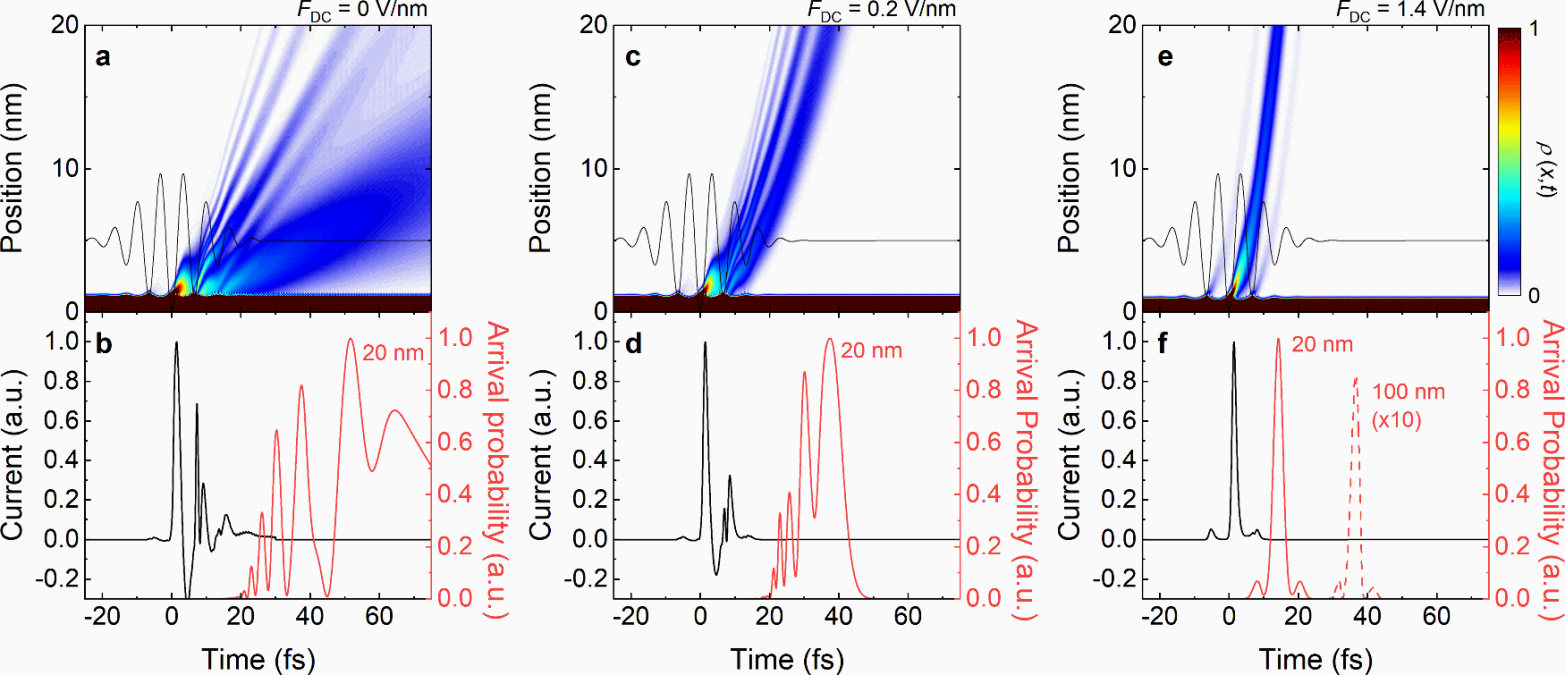
Simulated subcycle emission dynamics from biased gold nanotips, driven by 2000 nm pulses with a near-field strength of 2 V/nm (γ ≈ 3.7). (a) Emission dynamics for an unbiased gold tip. Electrons are emitted within two subsequent optical cycles, with a dominating contribution from the central cycle. The interference between direct and rescattered electrons results in a spatially and temporally structured electron density ρ(*x*, *t*). (b) Instantaneous emission current (black) and arrival probability (red) of the electrons in 20 nm distance. In the absence of a bias voltage, recollisions strongly modulate and stretch the electron pulse profile. (c, d) For a weak DC field of 0.2 V/nm, the electron wavepacket is accelerated away from the surface and recollisions are markedly suppressed. Hence, spatiotemporal modulation of ρ(*x*, *t*) is much reduced. This reduces the temporal spreading of the emitted electron beam upon propagation. (e, f) For a strong bias of 1.4 V/nm, electron acceleration away from the surface is so pronounced that the spatiotemporal overlap of electron beams emitted from subsequent cycles is negligible. Since electrons are predominantly emitted during the central cycle of the driving pulse, an isolated electron beam emerges that maintains its subcycle, 3 fs duration over a propagation distance of 100 nm.

This scenario changes markedly in the presence of a bias voltage (0.2 V/nm in Figure 4c,d) that accelerates the electrons away from the tip surface. This not only increases the average kinetic energy of the photoemitted electrons but also largely suppresses negative current contributions induced by near-field quivering. The pronounced intercycle interferences that appear without bias are suppressed. Consequently, the temporal dispersion of the generated electron beam is substantially reduced. Yet, the pulse duration after a few tens of nm propagation still increases to more than 20 fs.

Importantly, quiver oscillations, recollisions and the resulting intercycle interferences in the local electron density are basically completely suppressed by further increasing the bias to 1.4 V/nm (Figure 4e,f). Now, almost all electrons are generated in one half-cycle of the driving field. In these simulations, the strong bias reduces the photoemission nonlinearity to *s* = 5.2. This still is sufficient to restrict significant electron emission to the central half-cycle of the driving pulse. This generates an isolated emission burst with sub-fs duration near the tip apex. For such an isolated electron pulse, the pulse duration is mainly preserved upon propagation. The simulations predict a subcycle duration of ∼3 fs that is maintained after propagation distances of more than 100 nm.

In summary, we have investigated the emission of electron number states from a sharp gold tip in a highly nonlinear multiphoton regime. While single electrons are photoemitted in a seventh-order nonlinear process, the nonlinearity increases to 20 for electron triplets. Using few-cycle near-infrared driving pulses, this high nonlinearity results in interferometric electron autocorrelation traces for electron triplets that are mainly confined to a single emission peak with a width of 0.8 fs. While single electron emission shows a weak CEP dependence with 6% contrast, this increases markedly to 18% for the electron triplets. Together with the measured CEP modulation period of 2π, this points to a unidirectional emission model, even in the multiphoton photoemission regime. In this unidirectional model, electrons are photoemitted during the half-cycle of the driving field that accelerates the electrons away from the tip. This model accounts for the temporal profile of the EACs and the modulation period of the electron yield, yet overestimates the modulation contrast. Alternative bidirectional or envelope-driven emission models fail to reproduce the observed CEP modulation period.

Simulations of the time-dependent Schrödinger equation provide further insight into the emission dynamics. Without bias voltage, the quiver motion of the released electrons in the tip near field and electron recollisions govern the photoemission. This results in a temporally structured electron wavepacket that quickly disperses when propagating away from the tip. With increasing bias, quiver motion and recollisions are suppressed, resulting in an isolated subcycle electron beam with much reduced dispersion. For conditions mimicking our experiments, subcycle electron pulses are predicted even after propagation distances of up to 100 nm. This highly nonlinear multiphoton emission regime generates isolated, few-femtosecond electron pulses with comparatively narrow energy distribution. At the same time it prevents tip degradation, commonly observed in the strong-field regime. As such, it may be of interest for future low-energy electron microscopy experiments with ultrahigh temporal resolution.

## Data Availability

The data that support the findings of this study are available from the corresponding authors upon reasonable request.

## Supporting Information Available

Generation and characterization of CEP-stable near-infrared laser pulses, number state assignment, photoelectron spectra of electron number states, sub-Poissonian emission statistics of electron number states from gold tips, reduction of the emission nonlinearity by a strong bias voltage (PDF)

## References

[ref1] Gaida J. H.; Lourenço-Martins H.; Sivis M.; Rittmann T.; Feist A.; et al. García de Abajo, F. J.; Ropers, C. Attosecond electron microscopy by free-electron homodyne detection. Nat. Photonics 2024, 18, 509–515. 10.1038/s41566-024-01380-8.

[ref2] Nabben D.; Kuttruff J.; Stolz L.; Ryabov A.; Baum P. Attosecond electron microscopy of sub-cycle optical dynamics. Nature 2023, 619 (7968), 63–67. 10.1038/s41586-023-06074-9. 37258681

[ref3] Feist A.; Echternkamp K. E.; Schauss J.; Yalunin S. V.; Schäfer S.; Ropers C. Quantum coherent optical phase modulation in an ultrafast transmission electron microscope. Nature 2015, 521 (7551), 200–203. 10.1038/nature14463. 25971512

[ref4] Priebe K. E.; Rathje C.; Yalunin S. V.; Hohage T.; Feist A.; Schäfer S.; Ropers C. Attosecond electron pulse trains and quantum state reconstruction in ultrafast transmission electron microscopy. Nat. Photonics 2017, 11 (12), 793–797. 10.1038/s41566-017-0045-8.

[ref5] Ryabov A.; Thurner J. W.; Nabben D.; Tsarev M. V.; Baum P. Attosecond metrology in a continuous-beam transmission electron microscope. Science Advances 2020, 6 (46), eabb1393 10.1126/sciadv.abb1393. 33177078 PMC7673721

[ref6] Barwick B.; Flannigan D. J.; Zewail A. H. Photon-induced near-field electron microscopy. Nature 2009, 462 (7275), 902–906. 10.1038/nature08662. 20016598

[ref7] Park S. T.; Lin M.; Zewail A. H. Photon-induced near-field electron microscopy (PINEM): theoretical and experimental. New J. Phys. 2010, 12 (12), 123028 10.1088/1367-2630/12/12/123028.

[ref8] Quinonez E.; Handali J.; Barwick B. Femtosecond photoelectron point projection microscope. Rev. Sci. Instrum. 2013, 84 (10), 103710 10.1063/1.4827035. 24182122

[ref9] Müller M.; Paarmann A.; Ernstorfer R. Femtosecond electrons probing currents and atomic structure in nanomaterials. Nat. Commun. 2014, 5 (1), 5292 10.1038/ncomms6292. 25358554

[ref10] Bainbridge A. R.; Barlow Myers C. W.; Bryan W. A. Femtosecond few- to single-electron point-projection microscopy for nanoscale dynamic imaging. Structural Dynamics 2016, 3 (2), 023612 10.1063/1.4947098. 27158637 PMC4841798

[ref11] Latychevskaia T.; Wicki F.; Longchamp J.-N.; Escher C.; Fink H.-W. Direct Observation of Individual Charges and Their Dynamics on Graphene by Low-Energy Electron Holography. Nano Lett. 2016, 16 (9), 5469–5474. 10.1021/acs.nanolett.6b01881. 27536886

[ref12] Longchamp J.-N.; Rauschenbach S.; Abb S.; Escher C.; Latychevskaia T.; Kern K.; Fink H.-W. Imaging proteins at the single-molecule level. Proc. Natl. Acad. Sci. U. S. A. 2017, 114 (7), 1474–1479. 10.1073/pnas.1614519114. 28087691 PMC5321008

[ref13] Vogelsang J.; Talebi N.; Hergert G.; Wöste A.; Groß P.; Hartschuh A.; Lienau C. Plasmonic-Nanofocusing-Based Electron Holography. ACS Photonics 2018, 5 (9), 3584–3593. 10.1021/acsphotonics.8b00418.

[ref14] Vogelsang J.; Hergert G.; Wang D.; Groß P.; Lienau C. Observing charge separation in nanoantennas via ultrafast point-projection electron microscopy. Light: Science & Applications 2018, 7 (1), 55 10.1038/s41377-018-0054-5. PMC6107015 30839605

[ref15] Latychevskaia T.; Escher C.; Andregg W.; Andregg M.; Fink H.-W. Direct visualization of charge transport in suspended (or free-standing) DNA strands by low-energy electron microscopy. Sci. Rep. 2019, 9 (1), 8889 10.1038/s41598-019-45351-4. 31222124 PMC6586886

[ref16] Hergert G.; Wöste A.; Vogelsang J.; Quenzel T.; Wang D.; Gross P.; Lienau C. Probing Transient Localized Electromagnetic Fields Using Low-Energy Point-Projection Electron Microscopy. ACS Photonics 2021, 8 (9), 2573–2580. 10.1021/acsphotonics.1c00775.

[ref17] Wöste A.; Hergert G.; Quenzel T.; Silies M.; Wang D.; Groß P.; Lienau C. Ultrafast Coupling of Optical Near Fields to Low-Energy Electrons Probed in a Point-Projection Microscope. Nano Lett. 2023, 23 (12), 5528–5534. 10.1021/acs.nanolett.3c00738. 37278447 PMC10311584

[ref18] Hommelhoff P.; Sortais Y.; Aghajani-Talesh A.; Kasevich M. A. Field Emission Tip as a Nanometer Source of Free Electron Femtosecond Pulses. Phys. Rev. Lett. 2006, 96 (7), 077401 10.1103/PhysRevLett.96.077401. 16606139

[ref19] Ropers C.; Solli D. R.; Schulz C. P.; Lienau C.; Elsaesser T. Localized Multiphoton Emission of Femtosecond Electron Pulses from Metal Nanotips. Phys. Rev. Lett. 2007, 98 (4), 043907 10.1103/PhysRevLett.98.043907. 17358773

[ref20] Bormann R.; Gulde M.; Weismann A.; Yalunin S. V.; Ropers C. Tip-Enhanced Strong-Field Photoemission. Phys. Rev. Lett. 2010, 105 (14), 147601 10.1103/PhysRevLett.105.147601. 21230866

[ref21] Gulde M.; Schweda S.; Storeck G.; Maiti M.; Yu H. K.; Wodtke A. M.; Schäfer S.; Ropers C. Ultrafast low-energy electron diffraction in transmission resolves polymer/graphene superstructure dynamics. Science 2014, 345 (6193), 200–204. 10.1126/science.1250658. 25013072

[ref22] Ehberger D.; Hammer J.; Eisele M.; Krüger M.; Noe J.; Högele A.; Hommelhoff P. Highly Coherent Electron Beam from a Laser-Triggered Tungsten Needle Tip. Phys. Rev. Lett. 2015, 114 (22), 227601 10.1103/PhysRevLett.114.227601. 26196645

[ref23] Vogelgesang S.; Storeck G.; Horstmann J. G.; Diekmann T.; Sivis M.; Schramm S.; Rossnagel K.; Schäfer S.; Ropers C. Phase ordering of charge density waves traced by ultrafast low-energy electron diffraction. Nat. Phys. 2018, 14 (2), 184–190. 10.1038/nphys4309.

[ref24] Meier S.; Heimerl J.; Hommelhoff P. Few-electron correlations after ultrafast photoemission from nanometric needle tips. Nat. Phys. 2023, 19 (10), 1402–1409. 10.1038/s41567-023-02059-7.

[ref25] Haindl R.; Feist A.; Domröse T.; Möller M.; Gaida J. H.; Yalunin S. V.; Ropers C. Coulomb-correlated electron number states in a transmission electron microscope beam. Nat. Phys. 2023, 19 (10), 1410–1417. 10.1038/s41567-023-02067-7.

[ref26] Heimerl J.; Mikhaylov A.; Meier S.; Höllerer H.; Kaminer I.; Chekhova M.; Hommelhoff P. Multiphoton electron emission with non-classical light. Nat. Phys. 2024, 20, 945–950. 10.1038/s41567-024-02472-6.

[ref27] Meier S.; Heimerl J.; Hommelhoff P. Correlations in strong-field-emitted ultrashort electron pulses from metal needle tips. Laser Physics Letters 2024, 21 (4), 045301 10.1088/1612-202X/ad2b5a.

[ref28] Krečinić F.; Malter J.; Paarmann A.; Müller M.; Ernstorfer R. Point-projection microscopy of nano-localized photoemission currents at sub-40 fs time scales. arXiv 2018, 1803.01766 10.48550/arXiv.1803.01766.

[ref29] Schiffrin A.; Paasch-Colberg T.; Karpowicz N.; Apalkov V.; Gerster D.; Mühlbrandt S.; Korbman M.; Reichert J.; Schultze M.; Holzner S.; et al. Optical-field-induced current in dielectrics. Nature 2013, 493 (7430), 70–74. 10.1038/nature11567. 23222521

[ref30] Paasch-Colberg T.; Kruchinin S. Y.; Sağlam Ö.; Kapser S.; Cabrini S.; Muehlbrandt S.; Reichert J.; Barth J. V.; Ernstorfer R.; Kienberger R.; et al. Sub-cycle optical control of current in a semiconductor: from the multiphoton to the tunneling regime. Optica 2016, 3 (12), 1358–1361. 10.1364/OPTICA.3.001358.

[ref31] Ludwig M.; Aguirregabiria G.; Ritzkowsky F.; Rybka T.; Marinica D. C.; Aizpurua J.; Borisov A. G.; Leitenstorfer A.; Brida D. Sub-femtosecond electron transport in a nanoscale gap. Nat. Phys. 2020, 16 (3), 341–345. 10.1038/s41567-019-0745-8.

[ref32] Mattes M.; Volkov M.; Baum P. Femtosecond electron beam probe of ultrafast electronics. Nat. Commun. 2024, 15 (1), 1743 10.1038/s41467-024-45744-8. 38409203 PMC10897311

[ref33] Jagutzki O.; Cerezo A.; Czasch A.; Dorner R.; Hattas M.; Min H.; Mergel V.; Spillmann U.; Ullmann-Pfleger K.; Weber T.; et al. Multiple hit readout of a microchannel plate detector with a three-layer delay-line anode. IEEE Trans. Nucl. Sci. 2002, 49 (5), 2477–2483. 10.1109/TNS.2002.803889.

[ref34] Dombi P.; Pápa Z.; Vogelsang J.; Yalunin S. V.; Sivis M.; Herink G.; Schäfer S.; Groß P.; Ropers C.; Lienau C. Strong-field nano-optics. Rev. Mod. Phys. 2020, 92 (2), 025003 10.1103/RevModPhys.92.025003.

[ref35] Short R.; Mandel L. Observation of Sub-Poissonian Photon Statistics. Phys. Rev. Lett. 1983, 51 (5), 384–387. 10.1103/PhysRevLett.51.384.

[ref36] Schötz J.; Mitra S.; Fuest H.; Neuhaus M.; Okell W. A.; Förster M.; Paschen T.; Ciappina M. F.; Yanagisawa H.; Wnuk P.; et al. Nonadiabatic ponderomotive effects in photoemission from nanotips in intense midinfrared laser fields. Phys. Rev. A 2018, 97 (1), 013413 10.1103/PhysRevA.97.013413.

[ref37] Lemell C.; Tong X. M.; Krausz F.; Burgdörfer J. Electron Emission from Metal Surfaces by Ultrashort Pulses: Determination of the Carrier-Envelope Phase. Phys. Rev. Lett. 2003, 90 (7), 076403 10.1103/PhysRevLett.90.076403. 12633255

[ref38] Apolonski A.; Dombi P.; Paulus G. G.; Kakehata M.; Holzwarth R.; Udem T.; Lemell C.; Torizuka K.; Burgdörfer J.; Hänsch T. W.; et al. Observation of Light-Phase-Sensitive Photoemission from a Metal. Phys. Rev. Lett. 2004, 92 (7), 073902 10.1103/PhysRevLett.92.073902. 14995852

[ref39] Roudnev V.; Esry B. D. General Theory of Carrier-Envelope Phase Effects. Phys. Rev. Lett. 2007, 99 (22), 220406 10.1103/PhysRevLett.99.220406. 18233269

[ref40] Abel M. J.; Pfeifer T.; Jullien A.; Nagel P. M.; Bell M. J.; Neumark D. M.; Leone S. R. Carrier-envelope phase-dependent quantum interferences in multiphoton ionization. Journal of Physics B: Atomic, Molecular and Optical Physics 2009, 42 (7), 075601 10.1088/0953-4075/42/7/075601.

[ref41] Krüger M.; Schenk M.; Hommelhoff P. Attosecond control of electrons emitted from a nanoscale metal tip. Nature 2011, 475 (7354), 78–81. 10.1038/nature10196. 21734706

[ref42] Piglosiewicz B.; Schmidt S.; Park D. J.; Vogelsang J.; Groß P.; Manzoni C.; Farinello P.; Cerullo G.; Lienau C. Carrier-envelope phase effects on the strong-field photoemission of electrons from metallic nanostructures. Nat. Photonics 2014, 8 (1), 37–42. 10.1038/nphoton.2013.288.

[ref43] Förster M.; Paschen T.; Krüger M.; Lemell C.; Wachter G.; Libisch F.; Madlener T.; Burgdörfer J.; Hommelhoff P. Two-Color Coherent Control of Femtosecond Above-Threshold Photoemission from a Tungsten Nanotip. Phys. Rev. Lett. 2016, 117 (21), 217601 10.1103/PhysRevLett.117.217601. 27911540

[ref44] Yudin G. L.; Ivanov M. Y. Nonadiabatic tunnel ionization: Looking inside a laser cycle. Phys. Rev. A 2001, 64 (1), 013409 10.1103/PhysRevA.64.013409.

[ref45] Schenk M.; Krüger M.; Hommelhoff P. Strong-Field Above-Threshold Photoemission from Sharp Metal Tips. Phys. Rev. Lett. 2010, 105 (25), 257601 10.1103/PhysRevLett.105.257601. 21231628

[ref46] Park D. J.; Piglosiewicz B.; Schmidt S.; Kollmann H.; Mascheck M.; Lienau C. Strong Field Acceleration and Steering of Ultrafast Electron Pulses from a Sharp Metallic Nanotip. Phys. Rev. Lett. 2012, 109 (24), 244803 10.1103/PhysRevLett.109.244803. 23368330

[ref47] Seiffert L.; Paschen T.; Hommelhoff P.; Fennel T. High-order above-threshold photoemission from nanotips controlled with two-color laser fields. Journal of Physics B: Atomic, Molecular and Optical Physics 2018, 51 (13), 134001 10.1088/1361-6455/aac34f.

[ref48] Heimerl J.; Meier S.; Kirchner A.; Weitz T.; Hommelhoff P. Strong-field electron emission from gold needle tips. J. Vac. Sci. Technol. B 2023, 41 (5), 052803 10.1116/6.0002916.

[ref49] Kim H. Y.; Garg M.; Mandal S.; Seiffert L.; Fennel T.; Goulielmakis E. Attosecond field emission. Nature 2023, 613 (7945), 662–666. 10.1038/s41586-022-05577-1. 36697865 PMC9876796

[ref50] Dienstbier P.; Seiffert L.; Paschen T.; Liehl A.; Leitenstorfer A.; Fennel T.; Hommelhoff P. Tracing attosecond electron emission from a nanometric metal tip. Nature 2023, 616 (7958), 702–706. 10.1038/s41586-023-05839-6. 37100942

[ref51] Kakehata M.; Takada H.; Kobayashi Y.; Torizuka K.; Fujihira Y.; Homma T.; Takahashi H. Single-shot measurement of carrier-envelope phase changes by spectral interferometry. Opt. Lett. 2001, 26 (18), 1436–1438. 10.1364/OL.26.001436. 18049630

[ref52] Udem T.; Holzwarth R.; Hänsch T. W. Optical frequency metrology. Nature 2002, 416 (6877), 233–237. 10.1038/416233a. 11894107

[ref53] Petek H.; Ogawa S. Femtosecond time-resolved two-photon photoemission studies of electron dynamics in metals. Prog. Surf. Sci. 1997, 56 (4), 239–310. 10.1016/S0079-6816(98)00002-1.

[ref54] Johnson P. B.; Christy R. W. Optical Constants of the Noble Metals. Phys. Rev. B 1972, 6 (12), 4370–4379. 10.1103/PhysRevB.6.4370.

[ref55] Kittel C. Introduction to Solid State Physics; Wiley, 2004.

[ref56] Pan L. H.; Sullivan T. E.; Peridier V. J.; Cutler P. H.; Miskovsky N. M. Three-dimensional electrostatic potential, and potential-energy barrier, near a tip-base junction. Appl. Phys. Lett. 1994, 65 (17), 2151–2153. 10.1063/1.112775.

[ref57] Luo Y.; Zhou Y.; Zhang P. Few-cycle optical-field-induced photoemission from biased surfaces: An exact quantum theory. Phys. Rev. B 2021, 103 (8), 085410 10.1103/PhysRevB.103.085410.

[ref58] Eldar M.; Abo-Toame S.; Krüger M. Sub-optical-cycle electron pulse trains from metal nanotips. Journal of Physics B: Atomic, Molecular and Optical Physics 2022, 55 (7), 074001 10.1088/1361-6455/ac5e09.

