# Supplementary material for: Ultra-Nonlinear Subcycle Photoemission of Few-Electron States from Sharp Gold Nanotapers
Source: Nano Lett. 2024 Aug 20;24(35):11067–74. doi: 10.1021/acs.nanolett.4c03240 (PMC11378295; doi:10.1021/acs.nanolett.4c03240)
Supplement: Supplementary file 1 — nl4c03240_si_001.pdf [file nl4c03240_si_001.pdf]

Supporting Information for

Ultra-nonlinear Subcycle Photoemission of Few-Electron States from Sharp Gold Nanotapers

Germann Hergert*[1], Rasmus Lampe[1], Andreas Wöste[1], Christoph Lienau*[1]

[1] Institut für Physik and Center for Nanoscale Dynamics (CeNaD), Carl von Ossietzky Universität Oldenburg, Carl-von-Ossietzky Str. 9-11, 26129 Oldenburg, Germany

Correspondence to: germann.hergert@uol.de or christoph.lienau@uol.de

Content

1. Generation and characterization of CEP-stable near-infrared laser pulses 1
   1.1. Laser setup 1
   1.2. Pulse duration 2
   1.3. CEP stability 3
   1.4. EAC measurements 5
2. Number state assignment 5
3. Photoelectron spectra of electron number states 7
4. Sub-Poissonian emission statistics of electron number states from gold tips 8
5. Reduction of the emission nonlinearity by a strong bias voltage 10
6. References 11

1. Generation and characterization of CEP-stable near-infrared laser pulses

1.1. Laser setup

Near-infrared (NIR) pulses with a passively stabilized carrier envelope phase (CEP) are generated in a home-built noncollinear optical parametric amplifier (NOPA) with subsequent difference frequency generation (DFG)[1, 2]. The pulses are centered at 2000 nm and have a pulse duration of 14.6 fs (~2.3 cycles). A simplified scheme of the system used in this work is presented in Figure S1a. It has already been described in earlier work[3]. We use an Ytterbium-based fiber laser (Amplitude Systèmes, Tangerine) operating at 175 kHz with 35 W total output (200 µJ pulse energy) and 250 fs pulse duration at 1030 nm to pump the NOPA-DFG system.

Only a fraction of the total pump power is required to operate the entire setup. A fraction of 300 mW (1.7 µJ) is focused into a 4-mm thick Yttrium-Aluminum-Garnet (YAG) crystal to generate a white light (WL) supercontinuum down to 490 nm with a pulse energy of ~10 nJ.

The WL pulses are compressed to a duration of a few hundred fs using commercial chirped mirrors (LaserQuantum, DCM9) with 4 bounce pairs. These pulses serve as the seed for the NOPA process in a 2-mm thick Beta Barium Borate (BBO) crystal, cut at 22.3°. Another 65 µJ of the fundamental laser is used for second harmonic generation (SHG), yielding pulses around 515 nm (~40 µJ) that pump the NOPA-BBO. We amplify the spectral region between 630 nm and 720 nm from the WL and obtain an output of 2.3 µJ from the NOPA process. The third-order dispersion of the NOPA pulses is compensated using custom made chirp mirrors, applying a second- and third order dispersion of -42 fs^2 and -133 fs^3 per reflection. After 6 reflections, we measure a pulse duration of 12 fs for the NOPA pulses. This, however, does not yield the shortest DFG pulses, due to additional dispersion acquired after the NOPA. We therefore insert a tunable amount of ~6 mm fused silica as a wedge pair into the NOPA pulses to stretch them to a duration of ~110 fs. These pulses are combined with the fundamental laser (3.7 µJ) in a collinear alignment in a 1-mm thick BBO (23.4°) for the DFG-process. Fluctuations of the CEP in the fundamental laser pulses are directly transferred to the WL and NOPA pulses and will thus cancel each other in the DFG process, enabling passive CEP-stabilization[4, 5]. Before the DFG pulses are characterized, we use a silicon-coated FS-plate with 1-mm thickness as a filter (Si-Filter) to separate the DFG pulses from the NOPA and fundamental laser. The measured spectrum of the DFG pulses is shown in Figure S1b. It is centered around 2000 nm and ranges from 1650 nm to 2350 nm, with a pulse energy of 0.11 µJ.

1.2. Pulse duration

The pulse duration directly after the Si-Filter is measured by an interferometric frequency-resolved optical gating (IFROG) setup[6-8]. In the experiments described in the manuscript, the nanotip is located inside a vacuum chamber with a 1.5-mm thick Calcium fluoride (CaF_2) window. The pulses pass the CaF_2 window before inducing photoemission. To compensate the effect of this CaF_2 window on the IFROG measurement, we add a replica of this window into the beam path.

The DFG pulses with an electric field $E(t)$ are sent into a Michelson interferometer and split into a pair of identical pulses with variable time delay τ. This pulse pair is focused into a 20-µm thick BBO, cut at 20°, to generate a second harmonic field $E_{\mathrm{SHG}} = [E(t) + E(t-\tau)]^2$ which carries information about the DFG pulse duration. The spectrum of the SHG field is measured for various delays to obtain the IFROG trace[6]:

$$I_{\mathrm{IFROG}}(\omega,\tau) \propto \left| \int_{-\infty}^{\infty} [E(t) + E(t-\tau)]^2 e^{-i\omega t} \mathrm{d}t \right|^2$$

Using Fourier-filtering techniques we isolate the frequency-resolved optical gating (FROG) trace from the DC component of the IFROG trace, shown in Figure S1c as a function of the SHG wavelength[6]. In Figure S1d we show the resulting FROG trace using an established

retrieval algorithm[9]. We use the retrieved phase and the measured spectrum to calculate the electric field $E(t)$. Its intensity envelope (Figure S1e) reveals a pulse duration of 14.6 fs FWHM (full width at half maximum). This retrieved electric field is used for fitting the electron autocorrelations shown in Figure 3e-h in the main text.

1.3. CEP stability

The introduced NOPA-DFG scheme generates pulses with passive CEP stability[10, 11], as characterized in a common-path f-2f interferometer[12, 13]. The DFG pulses are focused into a 4-mm thick YAG crystal to generate CEP-preserving WL pulses with an octave spanning spectrum from 2000 nm down to 650 nm[14, 15]. By focusing the WL pulses into a 3-mm thick BBO crystal (23.4°), SHG is induced from the NIR part of the WL at 1400 nm. The generated second harmonic spectrally overlaps with the low-wavelength region of the WL around 700 nm. The SHG and WL pulses at 700 nm have opposite polarization, which prevents interferences between them. Therefore, a polarizer is introduced to project both pulses onto the same polarization direction. Both pulses are focused into a fiber spectrometer (Avantes, AvaSpec-ULS4096CL-EVO), with a fixed temporal delay between them. This leads to an interference pattern in their spectrum. The position of the interference fringes is sensitive to the CEP of the DFG pulses, because the CEP of the SHG pulses is doubled during their generation. An interferogram (Figure S1f) consisting of 3,000 spectra, each integrated over 500 µs (~90 laser pulses), is recorded within 5 minutes in the f-2f interferometer. By a Fourier-transform the CEP from each spectrum can be extracted. This is plotted in Figure S1g, revealing a standard deviation of 250 mrad over a time span of 5 minutes. For longer time scales the CEP shows stronger drifts that are shown in Figure S2h. An oscillation with a period of about 30 minutes can be seen, which are caused by similar oscillations in our laboratory's temperature.

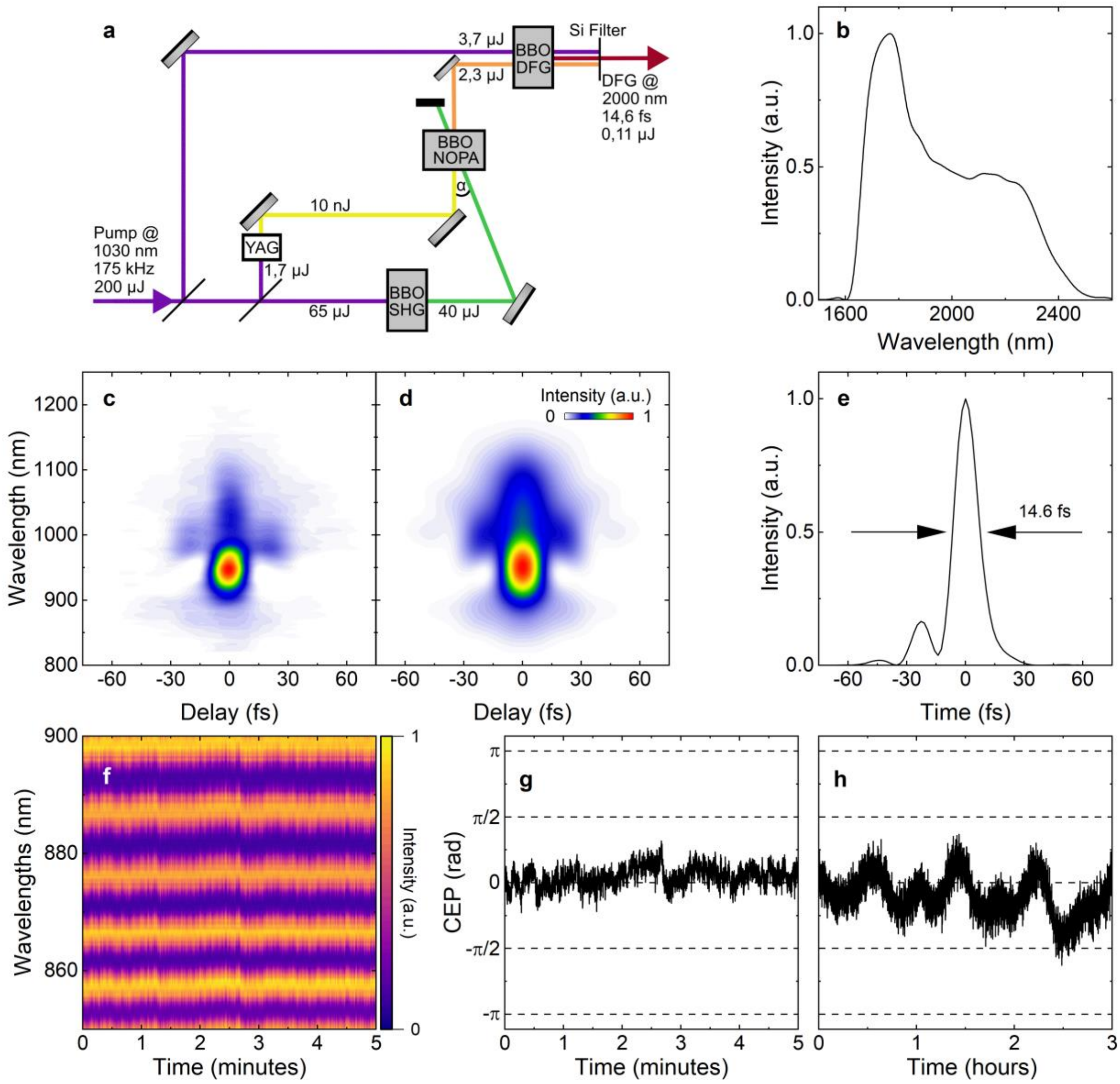

Supporting Figure S1. (a) Scheme of the setup for the generation of CEP-stable, few cycle near-infrared pulses via noncollinear parametric amplification (NOPA) and subsequent difference frequency generation (DFG). **(b)** Measured laser spectrum of the DFG pulses centered around 2000 nm, with a pulse energy of 0.11 µJ. **(c)** Measured spectrogram by interferometric frequency-resolved optical gating (IFROG) of the DFG pulses. **(d)** Retrieved IFROG trace from (c). **(e)** Intensity envelope of the DFG pulses at the location of the nanotaper in the experiments, revealing a pulse duration of 14.6 fs. **(f)** Measured interferogram of the DFG pulses in a f-2f interferometer. **(g)** Extracted CEP-stability from (f) over a duration of 5 minutes, with a standard deviation of 250 mrad. **(h)** Long-term CEP-stability over 3 hours, showing slow drifts in the CEP on a 30-minute time scale caused by the laboratory climate.

1.4. EAC measurements

Recording the electron autocorrelations (EAC) was performed by focusing a pair of two identical laser pulses with variable time delay onto the gold nanotaper and measuring the resulting electron spectra for each time delay. The pulse pair is created by separating the initial pulse with a 50:50 beam splitter with low group delay dispersion in a Michelson interferometer setup. After recombining both laser pulses with a second 50:50 beam splitter, each pulse has transmitted through ~1 mm of substrate, which however does not increase the pulse duration significantly.

We varied the time delay between the pulses by using a piezoelectric stage (PI, P-621.1CD) moving at a step size of 15 nm for a total range of 24 µm. During the measurement the stage is first moved to its new position and after a short pause time of 0.1 s the electron spectra are recorded for an integration time of 1 s. The temporal delay range translates to 160 fs at a resolution of 100 as. Already, this finite step size slightly broadens the measured EAC traces, resulting in a minor reduction of the extracted nonlinearity. In Figure 3 of the manuscript we report nonlinearities of 6.5 for the total yield and 6, 11 and 17 for $n = 1, 2, 3$ respectively. These values are systematically lower than the nonlinearities of 7.6 for the total yield and 7.7, 12.5 and 19.9 for $n = 1, 2, 3$ that we reported for the power-dependent measurements in Figure 1 of the manuscript. We presume that the step size of 100 as is too high to resolve the central peaks of the EAC traces that are quenched down to a FWHM of 800 as. Only a very limited amount of data points account for these central peaks which limits the ability to accurately resolve the form of the EAC traces, especially at the peak position. A smaller step size can improve the accuracy in extracting the nonlinearity from EAC measurements. We opted against in the interest to limit the recording time of an EAC trace. In the present experiments, the measurement of a single EAC trace took ~30 minutes. Due to the low event rate for the electron triplets we performed ten EAC measurements over a time span of ~5 hours and summed the spectra while distinguishing between electron number state. An exponentially decaying tail of background electrons has been subtracted for each electron spectrum. We further explain the emergence of this background and its subtraction from the electron spectra in Section 3.

2. Number state assignment

In the described experiments, the photoemission from the nanotaper is recorded using a time-of-flight delay-line detector (DLD) (RoentDek Handels GmbH, Hex80L). Based on characterization work performed in Refs. [16, 17] we expect that the low-energy electron detection efficiency of the detector exceeds 80%. We therefore expect that the finite quantum efficiency of the DLD plays a minor role for the results presented in our work.

This detection scheme gives access to the kinetic energy of each detected electron for each laser pulse. By making use of the multi-hit capability of the DLD, the kinetic energy of up to four electrons per pulse is retrieved. We describe the functionality of the DLD and the

energy measurement by following the path of the electrons through the experiment. The laser pulses are focused onto the nanotaper to emit a certain number n_0 of electrons per laser pulse. By applying a small bias voltage of -15 V to the tip, the electrons are accelerated towards the entrance of a drift tube, placed at a distance of ~1 cm from the tip. The tube has a length of ~18.6 cm length. Inside the drift tube, the electrons move ballistically in the field-free region towards the DLD that is attached at the end of the drift tube. The DLD consists of two multi-channel plates (MCP) with a diameter of 80 mm, ensued by three delay-line anodes that are rotated at 120° with respect to each other. An electron impinging on the MCPs is amplified to a charge cloud and subsequently hits the anodes. On each anode the multiplied electron cloud induces an electric pulse that propagates across the anode. The arrival times at the two ends of each anode are measured using a time discriminator. The time difference between these six arrival times is then used to calculate the point of impact on each delay line and the arrival time of the electron at the detector plane T_{D} relative to a laser trigger. In principle, only two delay lines, twisted against each other, are needed to calculate the impact position $(x_{\mathrm{D}}, y_{\mathrm{D}})$ in the detector plane for a single electron. The third delay line gives redundant information that is used to disentangle the positions of multiple electrons hitting the DLD for one laser pulse, so that we can measure T_{D}, x_{D} and y_{D} for each individual electron[18].

This information allows us to calculate the kinetic energy E_{kin} of each detected electron. For this we assume a fully ballistic motion of the electrons from the tip (placed at the origin) to the detector plane in a distance d. We take the impact positions as $\vec{r} = (x_{\mathrm{D}}, y_{\mathrm{D}}, d)$ and neglect the short acceleration path in front of the drift tube. The kinetic energy of each electron is then calculated by $E_{\mathrm{kin}} = \frac{1}{2} m \vec{r}^2 / (T_{\mathrm{D}} - t_{\mathrm{off}})^2$, where m is the electron mass. Here, t_{off} is a time delay that is used for a calibration of the energy axis of the detector. For this calibration, several electron spectra are measured at different bias voltages U and at low count rates ($\ll 1$ electron per pulse), to minimize strong-field effects. We then choose d and t_{off} in such a way that the low-energy cut-off of the spectra is always located at the electron energy qU, where q is the electron charge.

A typical electron spectrum at a laser power of 560 µW is shown in Figure S2a. We observe a 1-eV broad, distinct peak centered at around 15 eV, which is attributed to electron emission directly from the nanotaper. Additionally, a background of electron with lower energies is observed, that decays from 0 eV to higher energies. Our results suggest that these low-energy electrons may come from secondary electron scattering processes during the propagation towards the detector plane.

We then make use of the multi-hit capability of the DLD to assign a certain number state n, i.e., the number of detected electrons, to each laser pulse. Prior to the assignment, we consider only electrons with certain energies. If we only consider electrons with energies < 14 eV constituting the background in the spectrum, we obtain the power-dependent probability C_n to detect an n-electron event for each multielectron detection event in Figure

S2b. The colored dots show the event rate C_n for number states $n = 0 - 4$, respectively. For each power we integrated the electron yield over 10 seconds, corresponding to a total of 1.75×10^6 laser pulses at each power setting. The black dots show the average rate of detected electrons, given by $\langle C \rangle = \sum_{n=0}^{4} nC_n$. Higher number states with $n > 4$ are neglected because of their low probability at the considered laser powers. A linear fit through the data, plotted on a double-logarithmic scale (Figure S2b, solid lines), gives the order of the nonlinearity of the photoemission, $s_n = d\log(C_n)/d\log(P)$. For low laser powers, the plots show similar slopes of s of 7.3 ($n = 1$), 8.9 ($n = 2$), and 10.1 ($n = 3$) for the different number states. This changes if we only consider those electrons (Figure 2c), with energies > 14 eV, that are directly emitted from the nanotaper and reach the detector ballistically, without scattering losses. Again, the different event rates for the number states and the average yield are represented by the colored and black dots. Here we retrieve emission nonlinearities s of 7.7 (for $n = 1$), 12.5 (for $n = 2$), and 19.9 (for $n = 3$) that increase very strongly with increasing number state. This pronounced dependence of the nonlinearity on the number state at sufficiently low laser powers is a distinct signature that the emission occurs in the multiphoton photoemission regime.

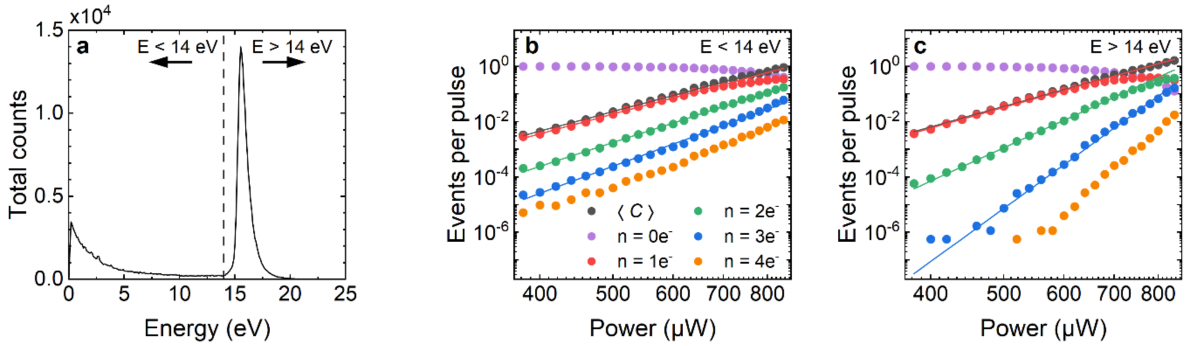

Supporting Figure S2. Multi-hit detection of multiphoton photoemission from gold nanotapers. **(a)** Kinetic energy spectrum of the total electron yield from a gold nanotaper biased at -15 V, recorded at a laser power of 560 μW. The spectrum shows a peak at 15 eV, attributed to electron emission directly from the tip, and a background of secondary low-energy electrons. **(b)** Power dependencies of the average detection rate (black dots) and the rates for different number states n (colored dots) when detecting only background electrons with energies < 14 eV. The extracted order of the emission nonlinearities s (solid lines) are 7.3 ($n = 1$), 8.9 ($n = 2$), and 10.1 ($n = 3$). **(c)** Power dependencies deduced for electrons with energies > 14 eV that are emitted and ballistically travel towards the detector. In this case, we observe nonlinearities s of 7.7 ($n = 1$), 12.5 ($n = 2$), 19.9 ($n = 3$) and 25 for $n = 4$.

3. Photoelectron spectra of electron number states

All measured photoelectron spectra presented in this work show a narrow peak with a FWHM of ~1.0 eV and are centered around ~15 eV, the bias voltage between tip and detector. Additionally, we observe a background peaked around 0 eV that is well modeled by a function that is exponentially decaying with energy which we have fitted to each electron spectrum. In Supporting Figure S3, we show the electron spectra for $n = 1, 2, 3$ from Figure 3 of the manuscript at a delay of 0 fs and a laser peak power of 620 μW before (a, b) and after (c, d) subtracting the background. In the cases of $n = 1$ and $n = 2$ the background

accounts in total for less than 1% and for less than 5% of the total counts for $n = 3$ at electron energies above 14 eV. While a broadening of the electron spectra with increasing n is observable for the raw spectra, this broadening is largely reduced after background correction and the spectra show similar FWHM for each electron number state. After background subtraction, all three spectra show very similar characteristic exponential decays on the high energy side of the spectrum that are usually taken as a sign of photoemission from a hot, non-equilibrium electron distribution[19]. Additionally, the total width of the electron spectra is affected by ponderomotive broadenings and the finite temporal width of the laser pulses. No attempts have been made to quantitatively model the lineshape of the emission spectra.

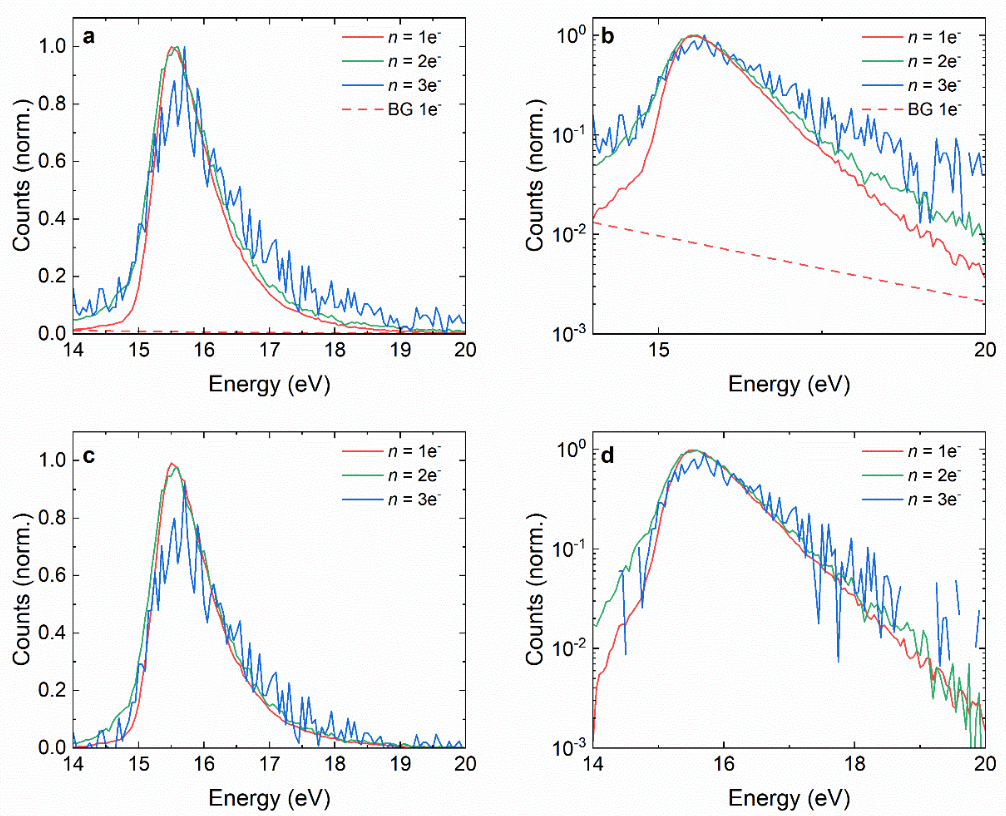

Supporting Figure S3. Kinetic energy spectra of different electron number states recorded at a peak power of 620 µW on a linear **(a, c)** and a logarithmic **(b, d)** scale. The spectra are taken from Fig. 3 at $\tau = 0$ fs. The FWHM is 1.2 eV and the spectra display a monoexponential decay at large energies. The raw spectra **(a, b)** contain electrons that arise from an exponentially decaying background of secondary electrons which has been indicated by the red dashed line for the $n = 1$ case. This background has been subtracted in the spectra shown in **(c, d)**.

4. Sub-Poissonian emission statistics of electron number states from gold tips

To obtain information about the photoemission statistics in our experiments, we analyze the power-dependent n-electron yields $p_{ex,n}(P)$ from Fig. S2(c) for n up to 4. In a first step, we deduced the average number of photoemitted electrons $\langle n \rangle(P) = \sum n p_{ex,n}(P)$. Up to the highest powers of our study, $\langle n \rangle$ scales with $P^{7.6}$ (filled blue circles in Figure S4a), demonstrating that all experiments are indeed performed in the multiphoton regime.

We then deduce the variance of the experimentally measured photoemission signals as $\langle \Delta n_{ex}^2 \rangle = \sum_{n=0}^{4} (n - \langle n \rangle)^2\, p_{ex,n}$. The data are shown as open black circles in Figure S4a. We compare them to the variance $\langle \Delta n_P^2 \rangle = \sum_{n=0}^{4} (n - \langle n \rangle)^2\, p_{P,n}(\langle n \rangle)$ (filled red triangles in Figure S4a) that is expected in the case of an ideal Poisson distribution $p_{P,n}(\langle n \rangle) = \langle n \rangle^n \exp(-\langle n \rangle)/n!$. The data show that for sufficiently high powers the measured variance is much smaller than expected for a Poisson distribution. We take this as a signature of sub-Poissonian statistics in the multi-electron emission regime.

To further quantify this, we deduce the Mandel Q Parameter[20], $Q = \langle (n - \langle n \rangle)^2 \rangle / \langle n \rangle$, which should be zero in the case of Poisson statistics, for which the variance equals the mean value. The results are reported in Figure S4b. Here, the experimental data Q_{ex}, deduced from the count rates of the first four number states, are shown as open black circles. The Q parameters Q_P, that are expected for a Poisson distribution for which only the first four number states are detected, are shown as red open circles in Figure S4b. The difference between both curves is marked as a shaded area.

For laser powers below 620 µW, the Mandel parameter is close to zero. Here, the average number of detected electrons is less than 0.2. Emission from higher number states makes a small contribution to the signal and we recover Poissonian statistics as expected for uncorrelated electron emission. For higher power, Q_{ex} decreases substantially below Q_P, a clear sign of sub-Poissonian noise statistics of the generated electron beam. Qualitatively, the results are in line with sub-Poissonian statistics observed in Refs. [21, 22]. In principle, the measured values for the Q-parameter may slightly be affected by the finite dead radius of the used delay-line detector[23]. We are therefore performing a more quantitative analysis of the energy correlations of the photoemitted electrons. This analysis will be presented in forthcoming work.

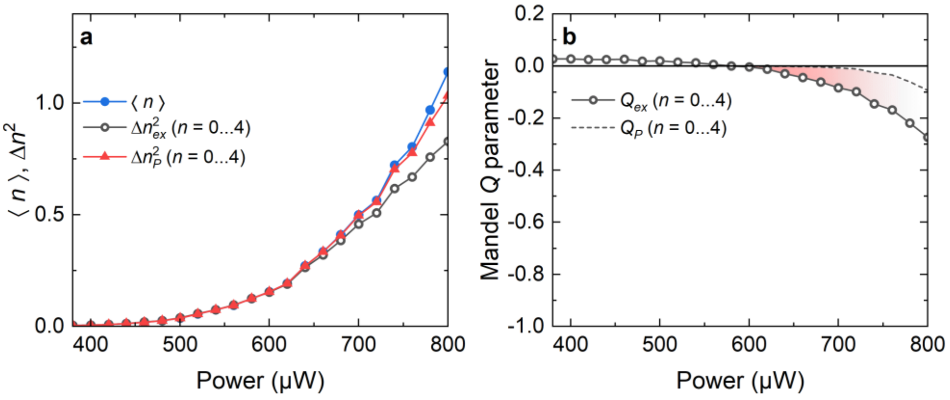

Supporting Figure S4. (a) Average number of photoemitted electrons $\langle n \rangle(P)$ (filled blue circles) as a function of laser power. The curve scales as $P^{7.6}$, giving evidence for multiphoton photoemission. The variance $\langle \Delta n_{ex}^2 \rangle$ of experimentally measured photoelectron yield (open black circles) is compared to the variance $\langle \Delta n_P^2 \rangle$ (filled red triangles) expected for a Poisson distribution when detecting only the lowest four number states. **(b)** Mandel Q parameter Q_{ex}, deduced from the experimental (open black circles) compared to the values Q_P for a Poisson distribution for which only the first four number states are detected. For higher power, Q_{ex} decreases substantially below Q_P, a sign of sub-Poissonian noise statistics of the generated electron beam.

5. Reduction of the emission nonlinearity by a strong bias voltage

The instantaneous photoemission current $C_{\text{inst}}(t)$ from biased metallic tips that is obtained from the simulated time-dependent Schrödinger equation (TDSE) simulations is depicted in Figure 4 of the main text. For high bias, almost all of the current is generated during the central half cycle of the pulse. Faint additional current peaks are also seen during the preceding and subsequent cycles. A close inspection shows that the preceding current pulse, for high bias, is slightly higher than for low bias. This is a consequence of the Schottky effect that lowers the work function by suppressing the binding potential of the electrons inside the metal[24-26]. This effectively lowers the emission nonlinearity and thus increases the pre- and postpulses in Figure 4e,f.

To demonstrate this Schottky effect and the resulting decrease in nonlinearity with increasing bias voltage, we simulate the effect of the local near-field amplitude on the emission current. We perform similar TDSE simulations as shown in Figure 4. Now, the local near-field amplitudes at the gold surface are varied between 1 V/nm and 10 V/nm ($\gamma = 0.7$). For each amplitude we calculate the total emission current $\int_{-\infty}^{\infty} C_{\text{inst}}(t)\,\mathrm{d}t$. The results are shown in Figure S5 (colored circles) for three different considered bias field strengths (0 V/nm, 0.2 V/nm, 1.4 V/nm) that have been used in the main manuscript. This decrease in nonlinearity provides evidence for the onset of the Schottky effect. For low near-field amplitudes, in the MPI regime, the curves show a nonlinear dependence on the field intensity $C_{\text{inst}}(t) \propto I^s$, with a slope s that decreases from 8.2 (0 V/nm), to 7.1 (0.2 V/nm) and 5.2 (1.4 V/nm) with increasing bias. For higher amplitude strong field effects set in[27]. The black dashed line in Figure S5 represents the near-field amplitude of 2 V/nm ($\gamma = 3.7$) that has been used for simulating the emission dynamics in Figure 4 of the main manuscript.

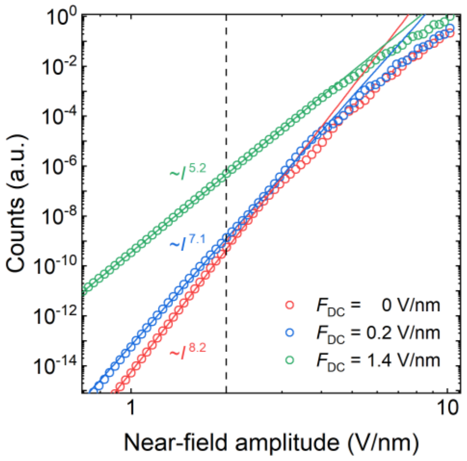

Supporting Figure S5. Time-dependent Schrödinger equation simulation of the intensity dependence of electron emission from biased gold surfaces. The total emission current is simulated for three different static bias voltages of 0 V/nm, 0.2 V/nm and 1.4 V/nm. The local near-field amplitude at the metal surface is varied between 1 and 10 V/nm. In the MPI regime, we observe a nonlinear dependence of the emission current on the laser intensity I of $I^{8.2}$, $I^{7.1}$ and $I^{5.2}$ for the three different bias voltages, respectively. The Schottky effect decrease the nonlinearity with increasing bias voltage.

6. References

(1) Manzoni, C.; Cerullo, G. Design criteria for ultrafast optical parametric amplifiers. *Journal of Optics* **2016**, *18* (10), 103501.

(2) Neuhaus, M.; Fuest, H.; Seeger, M.; Schötz, J.; Trubetskov, M.; Russbueldt, P.; Hoffmann, H. D.; Riedle, E.; Major, Z.; Pervak, V.; et al. 10 W CEP-stable few-cycle source at 2 µm with 100 kHz repetition rate. *Opt. Express* **2018**, *26* (13), 16074-16085.

(3) Wöste, A.; Hergert, G.; Quenzel, T.; Silies, M.; Wang, D.; Groß, P.; Lienau, C. Ultrafast Coupling of Optical Near Fields to Low-Energy Electrons Probed in a Point-Projection Microscope. *Nano Letters* **2023**, *23* (12), 5528-5534.

(4) Piglosiewicz, B.; Schmidt, S.; Park, D. J.; Vogelsang, J.; Groß, P.; Manzoni, C.; Farinello, P.; Cerullo, G.; Lienau, C. Carrier-envelope phase effects on the strong-field photoemission of electrons from metallic nanostructures. *Nature Photonics* **2014**, *8* (1), 37-42.

(5) Vogelsang, J.; Robin, J.; Piglosiewicz, B.; Manzoni, C.; Farinello, P.; Melzer, S.; Feru, P.; Cerullo, G.; Lienau, C.; Groß, P. High passive CEP stability from a few-cycle, tunable NOPA-DFG system for observation of CEP-effects in photoemission. *Opt. Express* **2014**, *22* (21), 25295-25306.

(6) Stibenz, G.; Steinmeyer, G. Interferometric frequency-resolved optical gating. *Opt. Express* **2005**, *13* (7), 2617-2626.

(7) Mascheck, M.; Schmidt, S.; Silies, M.; Yatsui, T.; Kitamura, K.; Ohtsu, M.; Leipold, D.; Runge, E.; Lienau, C. Observing the localization of light in space and time by ultrafast second-harmonic microscopy. *Nature Photonics* **2012**, *6* (5), 293-298.

(8) Zhong, J.-H.; Vogelsang, J.; Yi, J.-M.; Wang, D.; Wittenbecher, L.; Mikaelsson, S.; Korte, A.; Chimeh, A.; Arnold, C. L.; Schaaf, P.; et al. Nonlinear plasmon-exciton coupling enhances sum-frequency generation from a hybrid metal/semiconductor nanostructure. *Nature Communications* **2020**, *11* (1), 1464.

(9) Trebino, R.; Kane, D. J. Using phase retrieval to measure the intensity and phase of ultrashort pulses: frequency-resolved optical gating. *J. Opt. Soc. Am. A* **1993**, *10* (5), 1101-1111.

(10) Manzoni, C.; Cerullo, G.; De Silvestri, S. Ultrabroadband self-phase-stabilized pulses by difference-frequency generation. *Opt. Lett.* **2004**, *29* (22), 2668-2670.

(11) Cerullo, G.; Baltuška, A.; Mücke, O. D.; Vozzi, C. Few-optical-cycle light pulses with passive carrier-envelope phase stabilization. *Laser & Photonics Reviews* **2011**, *5* (3), 323-351.

(12) Kakehata, M.; Takada, H.; Kobayashi, Y.; Torizuka, K.; Fujihira, Y.; Homma, T.; Takahashi, H. Single-shot measurement of carrier-envelope phase changes by spectral interferometry. *Opt. Lett.* **2001**, *26* (18), 1436-1438.

(13) Udem, T.; Holzwarth, R.; Hänsch, T. W. Optical frequency metrology. *Nature* **2002**, *416* (6877), 233-237.

(14) Bellini, M.; Hänsch, T. W. Phase-locked white-light continuum pulses: toward a universal optical frequency-comb synthesizer. *Opt. Lett.* **2000**, *25* (14), 1049-1051.

(15) Baum, P.; Riedle, E.; Greve, M.; Telle, H. R. Phase-locked ultrashort pulse trains at separate and independently tunable wavelengths. *Opt. Lett.* **2005**, *30* (15), 2028-2030.

(16) Müller, A.; Djurić, N.; Dunn, G. H.; Belić, D. S. Absolute detection efficiencies of microchannel plates for 0.1–2.3 keV electrons and 2.1–4.4 keV Mg+ ions. *Review of Scientific Instruments* **1986**, *57* (3), 349-353.

(17) Fehre, K.; Trojanowskaja, D.; Gatzke, J.; Kunitski, M.; Trinter, F.; Zeller, S.; Schmidt, L.; Stohner, J.; Berger, R.; Czasch, A.; et al. Absolute ion detection efficiencies of microchannel plates and funnel microchannel plates for multi-coincidence detection. *Review of Scientific Instruments* **2018**, *89*, 045112.
(18) Jagutzki, O.; Cerezo, A.; Czasch, A.; Dorner, R.; Hattas, M.; Min, H.; Mergel, V.; Spillmann, U.; Ullmann-Pfleger, K.; Weber, T.; et al. Multiple hit readout of a microchannel plate detector with a three-layer delay-line anode. *IEEE Transactions on Nuclear Science* **2002**, *49* (5), 2477-2483.
(19) Petek, H.; Ogawa, S. Femtosecond time-resolved two-photon photoemission studies of electron dynamics in metals. *Progress in Surface Science* **1997**, *56* (4), 239-310.
(20) Short, R. W.; Mandel, L. Observation of Sub-Poissonian Photon Statistics. *Physical Review Letters* **1983**, *51*, 384-387.
(21) Haindl, R.; Feist, A.; Domrose, T.; Moller, M.; Gaida, J. H.; Yalunin, S. V.; Ropers, C. Coulomb-correlated electron number states in a transmission electron microscope beam. *Nature Physics* **2023**.
(22) Meier, S.; Heimerl, J.; Hommelhoff, P. Few-electron correlations after ultrafast photoemission from nanometric needle tips. *Nature Physics* **2023**.
(23) Knipfer, M.; Meier, S.; Volk, T.; Heimerl, J.; Hommelhoff, P.; Gleyzer, S. Deep learning-based spatiotemporal multi-event reconstruction for delay line detectors. *Machine Learning: Science and Technology* **2024**, *5* (2), 025019.
(24) Schottky, W. Über Katalyse durch Strahlung und strahlungsähnliche Prozesse. *Zeitschrift für Physik* **1914**, *15* (1), 872-874.
(25) Fowler, R. H.; Nordheim, L. Electron emission in intense electric fields. *Proceedings of the Royal Society of London. Series A, Containing Papers of a Mathematical and Physical Character* **1928**, *119* (781), 173-181.
(26) Ropers, C.; Solli, D. R.; Schulz, C. P.; Lienau, C.; Elsaesser, T. Localized Multiphoton Emission of Femtosecond Electron Pulses from Metal Nanotips. *Physical Review Letters* **2007**, *98* (4), 043907.
(27) Dombi, P.; Pápa, Z.; Vogelsang, J.; Yalunin, S. V.; Sivis, M.; Herink, G.; Schäfer, S.; Groß, P.; Ropers, C.; Lienau, C. Strong-field nano-optics. *Reviews of Modern Physics* **2020**, *92* (2), 025003.
